# Functional Analyses of the Crohn’s Disease Risk Gene *LACC1*

**DOI:** 10.1371/journal.pone.0168276

**Published:** 2016-12-13

**Authors:** Ghazaleh Assadi, Liselotte Vesterlund, Ferdinando Bonfiglio, Luca Mazzurana, Lina Cordeddu, Danika Schepis, Jenny Mjösberg, Sabrina Ruhrmann, Alessia Fabbri, Vladana Vukojevic, Piergiorgio Percipalle, Florian A. Salomons, Jurga Laurencikiene, Leif Törkvist, Jonas Halfvarson, Mauro D’Amato

**Affiliations:** 1 Department of Biosciences and Nutrition, Karolinska Institutet, Stockholm, Sweden; 2 Center for Infectious Medicine, Department of Medicine Huddinge, Karolinska Institutet, Stockholm, Sweden; 3 Rheumatology unit, Department of Medicine Solna, Karolinska University Hospital, Karolinska Institutet, Stockholm, Sweden; 4 Department of Clinical Neuroscience, Karolinska Institutet, Stockholm, Sweden; 5 Department of Therapeutic Research and Medicines Evaluation, Istituto Superiore di Sanità, Rome, Italy; 6 Biology Program, New York University Abu Dhabi, Abu Dhabi, United Arab Emirates; 7 Department of Molecular Biosciences, The Wenner-Gren Institute, Stockholm University, Stockholm, Sweden; 8 Department of Cell and Molecular Biology, Karolinska Institutet, Stockholm, Sweden; 9 Lipid laboratory, Department of Medicine Huddinge, Karolinska Institutet, Stockholm, Sweden; 10 Gastrocentrum, Karolinska University Hospital, Stockholm, Sweden; 11 Department of Gastroenterology, Faculty of Medicine and Health, Örebro University, Örebro, Sweden; 12 BioDonostia Health Research Institute, San Sebastian and IKERBASQUE, Basque Foundation for Science, Bilbao, Spain; Bambino Gesù Children's Hospital, ITALY

## Abstract

**Background:**

Genetic variation in the *Laccase (multicopper oxidoreductase) domain-containing 1* (*LACC1*) gene has been shown to affect the risk of Crohn’s disease, leprosy and, more recently, ulcerative colitis and juvenile idiopathic arthritis. *LACC1* function appears to promote fatty-acid oxidation, with concomitant inflammasome activation, reactive oxygen species production, and anti-bacterial responses in macrophages. We sought to contribute to elucidating *LACC1* biological function by extensive characterization of its expression in human tissues and cells, and through preliminary analyses of the regulatory mechanisms driving such expression.

**Methods:**

We implemented Western blot, quantitative real-time PCR, immunofluorescence microscopy, and flow cytometry analyses to investigate fatty acid metabolism-immune nexus (FAMIN; the *LACC1* encoded protein) expression in subcellular compartments, cell lines and relevant human tissues. Gene-set enrichment analyses were performed to initially investigate modulatory mechanisms of *LACC1* expression. A small-interference RNA knockdown *in vitro* model system was used to study the effect of FAMIN depletion on peroxisome function.

**Results:**

FAMIN expression was detected in macrophage-differentiated THP-1 cells and several human tissues, being highest in neutrophils, monocytes/macrophages, myeloid and plasmacytoid dendritic cells among peripheral blood cells. Subcellular co-localization was exclusively confined to peroxisomes, with some additional positivity for organelle endomembrane structures. *LACC1* co-expression signatures were enriched for genes involved in peroxisome proliferator-activated receptors (PPAR) signaling pathways, and PPAR ligands downregulated FAMIN expression in *in vitro* model systems.

**Conclusion:**

FAMIN is a peroxisome-associated protein with primary role(s) in macrophages and other immune cells, where its metabolic functions may be modulated by PPAR signaling events. However, the precise molecular mechanisms through which FAMIN exerts its biological effects in immune cells remain to be elucidated.

## Introduction

The two major forms of inflammatory bowel disease (IBD), Crohn’s disease (CD) and ulcerative colitis (UC), are chronic inflammatory diseases of unknown etiology, affecting around 2.5 million of Europeans with an incidence that is increasing worldwide [1]. Both CD and UC represent complex immunologically mediated diseases that are thought to occur due to a dysregulated immune response to commensal gut microbiota in a genetically predisposed host [2,3].

Recent genome-wide association studies (GWAS) have identified 200 genomic regions (risk loci) associated with IBD [4,5], and highlighted the importance of innate immune interactions with the intestinal flora, the regulation of immune functions, the maintenance of gut epithelial barrier, and autophagy [4]. Despite the gained knowledge and advances in IBD genetics, the translation of research findings into functional insight and clinical applications has been difficult, primarily hampered by the fact that many causative genes and their respective pathophysiological roles are still to be elucidated. Ultimately, these genes represent important targets, as their functional characterization may pave the way to new intervention strategies and therapeutic exploitation.

Single nucleotide polymorphisms (SNPs) in the gene *Laccase (multicopper oxidoreductase) domain-containing 1 (LACC1*, previously known as *C13orf31)* have consistently been shown to associate with CD genetic risk in a number of GWA studies and their meta-analyses [4–6]. Of note, *LACC1* SNPs represent strong genetic risk factors not only for CD but also for leprosy [4–10], and we have recently reported the occurrence of *LACC1* common risk variants in UC and juvenile idiopathic arthritis (JIA) patients [11]. This is in addition to previous studies reporting a rare *LACC1* missense mutation (Cys284Arg) in monogenic forms of early-onset CD (EOCD) and systemic JIA (sJIA) [12,13]. Collectively, these observations suggest that *LACC1* is involved in a series of immune-mediated diseases with several clinical features in common, and that it may play an important role in inflammation and/or the response to infectious insults.

The protein encoded (fatty acid metabolism-immune nexus; FAMIN) by the *LACC1* gene is unique in that it shows no similarity to other mammalian proteins, but contains a C-terminal domain homologous to bacterial proteins of the multi-copper polyphenol oxidoreductases (PO) and laccase family. These are enzymes that catalyze the oxidation of aromatic substrates [14], and have been shown to be key components of the insect immune system [15]. Recently, we reported an initial characterization of *LACC1* function in *in vitro*, *in vivo* and *ex vivo* model systems, and implicated the encoded protein in inflammasome activation, mitochondrial and nicotinamide adenine dinucleotide phosphate (NADPH) oxidase-dependent reactive oxygen species (ROS) production, and bactericidal activity of macrophages. We showed that all of these defence mechanisms are controlled by FAMIN-dependent fatty-acid oxidation (FAO) [16].

In this study, we provide additional insight into FAMIN function with 1) a comprehensive characterization of FAMIN expression in human cell lines, tissues and immune cells; 2) a thorough characterization of FAMIN subcellular localization; 3) an analysis of *LACC1* co-expression with other genes, and its regulation by peroxisome proliferator-activated receptor (PPAR) signaling pathways.

## Materials and Methods

### Cell lines and transfections

The human cell line THP-1 (monocytic leukemia) was maintained in complete RPMI-1640 medium (GIBCO Invitrogen Life Technologies) supplemented with 10% FBS, 100 U of penicillin, 100 μg/ml streptomycin and 0.05 mM β-mercaptoethanol. In order to induce macrophage differentiation, THP-1 cells were plated on 6-well plates (2.0 x 10^6^ cells/well) and incubated with 100 ng/ml phorbol 12-myristate 13-acetate (PMA; Sigma-Aldrich, P1585) overnight. The PMA containing media was replaced after 24hrs with fresh media and the cells were incubated for an additional 24hrs. Fresh media containing increasing concentrations (0.1, 0.5, 1 or 2 μg) of LPS (InvivoGen, tlrl-smlps) was added to the cells following 24hrs incubation, upon which RNA and proteins were extracted. For analysis of the regulation of FAMIN expression cell culture media was changed, after PMA differentiation, either to fresh media or to media containing different concentration of PPAR ligands WY14643 (Sigma-Aldrich, C7081) or rosiglitazone (Sigma-Aldrich, R2408) as indicated, and cells were incubated for 6 and 24hrs, followed by protein extraction. The HeLa (cervix epithelial adenocarcinoma) cell line was maintained in DMEM (GIBCO Invitrogen Life Technologies), low glucose, supplemented with 10% FBS, 100 U of penicillin and 100 μg/ml streptomycin. For *LACC1* transfection experiments, HeLa cells were plated on 6-well plates (4 x 10^5^ cells/well) and incubated overnight. Transfections were performed at 70–90% confluency with either 1 μg empty vector (GFP from Lonza, pmaxGFP DNC-00051-14) or 0.5 μg empty vector in combination with 0.5 μg of different LACC1 vector constructs (LACC1-wt with a pReceiver-M02 vector, GFP-LACC1-wt with a pReceiver-M29 vector from GeneCopoeia, EX-T6419-M02 and EX-T6419-M29, respectively) per 100 μl transfection complex using 3 μl X-tremeGENE 9 DNA Transfection Reagent (Roche, 06365787001) in 100 μl Opti-MEM^®^ I Reduced-Serum Medium (GIBCO Invitrogen Life Technologies). The 100 μl transfection complexes were incubated 15 minutes at room temperature (RT) before added to each well followed by overnight incubation before protein extraction.

### Western blot

THP-1 or HeLa cells, differentiated, transfected and/or stimulated as described, were washed in ice-cold PBS and lysed in RIPA buffer (25 mM Tris-HCl pH 8.0, 150 mM NaCl, 0.1% SDS, 1% sodium deoxycholate, 1% Triton X-100) containing protease inhibitor cocktail tablet (Roche, 11836153001). Cells were incubated on ice for 10 minutes then homogenized by shearing 10 times through a 21-gauge needle. The protein lysate was centrifuged for 15 minutes at 4°C at 3,000 g and protein supernatants collected into a freshly chilled tube to remove cellular debris. Protein concentration was determined with Bradford colorimetric assay (BCA; Thermo Fisher Scientific, 23225) and 3 μg of proteins was loaded onto 10% SDS polyacrylamide gels (Thermo Fisher Scientific, NP0301). Using a Trans-Blot SD semi-dry transfer system (Bio-Rad), proteins were transferred onto PVDF membranes (GE Healthcare, RPN303F) followed by 1hrs incubation in 5% milk/PBS-T solution to block non-specific binding, and then overnight at 4°C in 2.5% milk/PBS-T with primary antibodies. Membranes were then washed with PBS-T, followed by 2hrs incubation with corresponding secondary antibody in 2.5% milk/PBS-T at RT before final PBS-T wash and autoradiograph development using Amersham^™^ ECL Select^™^ Western Blotting Detection Reagent (GE Healthcare, RPN2235). Densitometric analysis was performed with the imaging software ImageJ using β-actin for normalization. Expression levels were calculated as fold changes, respective to control (PMA treated) cells set as reference value = 1. Values are reported as relative intensity (RI).

### Immunofluorescence staining

THP-1 cells were seeded on glass coverslips and differentiated into macrophages overnight, before fixation with 4% formaldehyde (FA; Sigma-Aldrich, F1635) containing 0.2% Triton X-100 (Sigma-Aldrich, T9284) for 10 minutes and permeabilized with 1% Triton X-100 for 10 minutes. Fixed cells were washed with PBS and non-specific binding was blocked with 0.5% milk in PBS before incubation with primary antibodies in 0.25% milk in PBS for 1hr. Unbound antibody was removed by washing with PBS and secondary antibody in 0.25% milk in PBS was added and incubated for 30 minutes. Unbound secondary antibody was removed and cells were washed with PBS and stained with DAPI (Sigma-Aldrich, D9542) for 1 minute. Coverslips were washed and mounted with Mowiol mountant (Sigma-Aldrich, 324590). Fluorescence was visualized with a DeltaVision Spectris Deconvolution Microscope (GE Healthcare Life Sciences) with a 100x PlanApo 1.35 objective (Olympus). Images were deconvolved with the SoftWoRx software (GE Healthcare Life Sciences). For the experiments studying the effect of PPAR ligands on FAMIN expression, the number of peroxisomes was calculated using a custom ImageJ macro on 10 images per condition and triplicate experiments. Values were normalized according to the controls (untreated cells).

### Antibodies used for immunofluorescence and Western blot

The following antibodies were used: LACC1 E-12 (anti-FAMIN; sc-376231), E-7 (sc-374553), H-6 (sc-376064) from Santa Cruz; anti-β actin (ab6276) and anti-PMP70 (ab3421) from Abcam; anti-β-tubulin (2128), anti-calnexin (2679), anti-calreticulin (12238), anti-catalase (12980), anti-caveolin-2 (8522), anti-CENP-A (2186), anti-COX-IV (4850), anti-EEA1 (3288), anti-fibrillarin (2639), anti-histoneH3 (4499), anti-LAMP1 (9091), anti-LC3B (2868), anti-NUP98 (2598), anti-PDI (2446), anti-Rab5 (3547) and anti-syntaxin-6 (2869) from Cell Signaling; anti-ABCD3 (HPA032027) from Sigma Aldrich.

### Laccase assay

FAMIN laccase enzymatic activity was measured with recombinant FAMIN purified protein (C-terminal MYC/DDK tagged; OriGene, TP307834) using a standard spectrophotometric laccase assay and different oxidation substrates (Sigma-Aldrich) 2,6-dimethoxyphenol (DMP; D135550), guaiacol (G5502), L-DOPA (D9628) and syringaldazine (SGZ; S7896). The assays were read in a plate spectrophotometer (Tecan Infinite^®^200 PRO) at 37°C in 100 mM sodium acetate buffer pH 5.0 with a final concentration of 1 mM for all substrates except for SGZ (25 μM) in a total volume of 200 μl/well. Fourteen nM of the fungal laccase from *Trametes versicolor* (Sigma-Aldrich, 38429) was used as a positive control, 14 nM of recombinant FAMIN was used as the tested protein and substrate blanks (negative control) were used in all assays. For all the reactions 150 μl of 100 mM sodium acetate solution was mixed with 30 μl of 14 nM *T*. *vesicolor* laccase or the recombinant FAMIN protein and 20 μl of substrate. The laccase activity was determined by measuring absorbance change every 3 minutes during 30–60 minutes.

### Quantitative real-time PCR

Total RNA was extracted from lysed THP-1 cells using the RNeasy Mini-kit (QIAGEN Inc, Hilden, Germany) and reverse transcribed to cDNA using SuperScriptTM III Reverse Transcriptase reagents (Invitrogen, Carlsbad, CA, USA), according to manufacturer’s instructions. *LACC1* mRNA expression levels were measured by quantitative real-time PCR (qRT-PCR) using TaqMan gene expression assays (Thermo Fisher Scientific, 4369016) and the 7500 Fast Real-Time PCR system (Applied Biosystems). qRT-PCR reactions were performed in triplicate on each sample with TaqMan assays Hs00403037_m1 for *LACC1* and Hs99999905_m1 for *GAPDH*. After normalization to the endogenous housekeeping gene *GAPDH*, *LACC1* level of expression in each sample was determined by the comparative C_T_ method of relative quantification, and expressed in arbitrary units relative to non-stimulated cells. Multiple tissue cDNA panels (MTC; Clontech, 636746 and 636748) were also used for measurement of *LACC1* mRNA expression in human tissues (as described above). These MTC panels contained cDNA from the human digestive and immune systems.

### Flow cytometry of human peripheral blood-derived cells

Peripheral blood mononuclear cells (PBMCs) were isolated from buffy coat from healthy donors using Lymphoprep (Stem Cell Technologies). Granulocytes were simultaneously isolated during Lymphoprep gradient centrifugation. Cells were then treated with 4% dextran, followed by hypotonic lysis to remove erythrocytes excess. Human PBMCs and granulocytes were stained for cell surface and intracellular markers directly after separation. Cells were multi-color stained using monoclonal antibodies (BD Bioscience, Biolegend and Milteny Biotec) specific for human CD3-PB (clone UCHT1, 30041), CD11c-PE/Cy7 (clone 3.9, 301608), CD14-BV510 (clone M5E2, 301842), CD15-PE (clone HI98, 301905), CD16-PB (clone 3G8, 558122), CD19-APC/Cy7 (clone SJ25C1, 557791), CD303-PE (BCDA-2) (clone AC144, 130-090-511), HLA-DR-FITC (clone TU36, 555560) and analyzed by fluorescence-activated cell sorting (FACS). For the intracellular staining of FAMIN cells were fixed in 4% FA for 15 minutes and subsequently permeabilized 15 minutes with 1% Triton X-100 at RT (as optimized for immunofluorescence). The cells where then stained in permeabilization buffer (Biolegend, 421002) using the FAMIN E-12 antibody labeled with Alexa Fluor^®^ 647 antibody labeling kit from Qiagen (A-20186). Data were acquired on a Gallios flow cytometer (Beckman Coulter, Inc.) calibrated with single-stained beads (BD^™^ CompBead, 552843) and single fluorochrome stained cells, and analyzed with Kaluza^®^ Flow Analysis Software (Beckman Coulter). “Fluorescence-minus-one” (FMO) controls were included to determine the level of nonspecific staining and auto-fluorescence associated with subsets of cells in each channel. Different gating strategies were set based on FMO for FAMIN.

### Small interfering RNA mediated knockdown

THP-1 cells were transfected with ON-TARGETplus SMARTpool targeting human *LACC1* (L-015653-02, Thermo Fisher Scientific, Lafayette CO) or Non-targeting Control Pool (D-001810-10) small interfering RNA (siRNA) using the NEON transfection system (Life Technologies, Carlsbad, CA) according the manufactures instructions. The ON-TARGETplus SMARTpool targeting *LACC1* consisted of a pool of 4 *LACC1*-directed siRNA sequences; CGAAAGAUGUAGAGGUUUU (J-015653-17), CUAGAUAAGAGGCGAUCAA (J-015653-18), AGACAUUGUUGUUGUACUU (J-015653-19) and GGAGAAAUUUUACCGAAUA (J-015653-20). In short, for each transfection, 1 μl (0.05 μM) of *LACC1* or control siRNA was mixed with 2 x 10^5^ cells in 9 μl buffer R. Cells were transfected with two 1400V pulses for 20 ms/puls and plated in media without antibiotics. To induce macrophage differentiation, cells were incubated for 48hrs with 100 ng/ml PMA, washed, and incubated for further 24hrs with fresh media. For PPAR ligand experiments, after PMA treatment for 48hrs, the media was changed into 100 μM WY14643 and the cells were incubated for 24hrs, followed by protein extraction. FAMIN knockdown (KD) efficiency was evaluated by Western blot (WB) as described above.

### *In silico* analyses

The Human Protein Atlas (HPA; http://www.proteinatlas.org/) [17] and the publicly available Genevisible search engine (https://genevisible.com) [18] were exploited to identify cell lines and tissues where *LACC1* is expressed. For the analysis of *LACC1* co-expression pathways, we adopted a 2-step strategy based on Genevestigator (http://www.genevestigator.com) [18], which is a high-performance search engine for gene expression, integrating thousands of manually curated public microarray data and RNA sequencing experiments. First, a list of genes showing co-regulated expression with *LACC1* was obtained by screening 35.000 gene expression microarray experiments, selecting genes with a correlation coefficient r ≥ 0.35 across the perturbations dataset. This gene list was then fed into the gene enrichment analysis pipeline of the gene functional classification tool Enrichr (http://amp.pharm.mssm.edu/Enrichr/) [19] in order to identify *LACC1*-relevant enriched biological pathways according to gene ontology terms (GO; http://www.geneontology.org) [20].

## Results

### Characterization of *LACC1* mRNA expression

**Fig 1:**
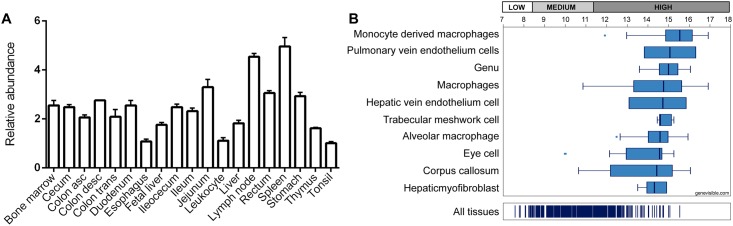
*LACC1* mRNA expression in immune-related and gastrointestinal human tissues. (A) qRT-PCR analysis of *LACC1* relative expression in a panel of human tissues identifies spleen and lymph nodes as tissues with higher *LACC1* expression. Reactions were done in triplicates. (B) *LACC1* mRNA expression data extracted from Genevisible (https://genevestigator.com/gv/).

To characterize *LACC1* product distribution in different human tissues and cells, we analyzed its mRNA expression patterns by qRT-PCR and retrieved publicly available data from web bio-sources. First, we undertook a qRT-PCR analysis using commercially available human tissue cDNA panels (MTC blots, see methods) to investigate gene expression in relation to human digestive and immune systems. The results showed that *LACC1* is ubiquitously expressed in a wide range of tested tissues, with relatively higher expression levels in immune-related tissues such as lymph nodes and spleen (Fig 1A). These results could be refined by retrieving additional publicly available expression data from the Genevisible web bio-source, which indicated highest *LACC1* mRNA expression in macrophages and monocyte-derived macrophages among other tissues (Fig 1B).

### Characterization of FAMIN protein expression

#### Characterization of FAMIN antibodies

**Fig 2:**
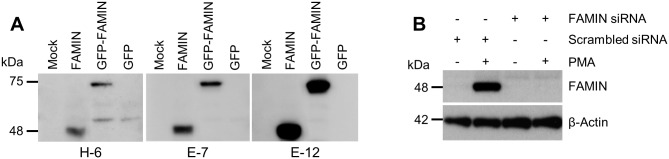
Characterization of anti-FAMIN antibodies and FAMIN expression in HeLa and THP-1 cell lines. (A) Anti-FAMIN mouse monoclonal antibodies (H-6, E-7 and E-12) were used in WB analyses of cell extracts from HeLa cells untransfected (mock) or transfected with FAMIN, GFP-FAMIN or GFP (control vector). (B) FAMIN expression is induced upon PMA differentiation of THP-1 cells (lanes 1–2 cells transfected with scrambled-control siRNA). Complete knockdown of FAMIN expression was obtained upon transfection of THP-1 cells with siRNA targeting *LACC1* transcript (lanes 3–4). β-actin was used for equal loading control. Data are representative of three independent experiments.

The HPA and Genevisible web repositories were screened to identify at least one *LACC1*-negative and one *LACC1*-positive human cell line suitable for *in vitro* experiments. First, we selected the *LACC1*-negative epithelial HeLa cell line and the *LACC1*-positive monocytic THP-1 cell line, which show endogenous expression and FAMIN-upregulation upon stimulation with PMA and LPS. We then sought to confirm this evidence by studying FAMIN expression in these cell lines by means of WB and qRT-PCR analyses. First, we set to confirm the specificity of three commercially available anti-FAMIN mouse monoclonal antibodies, namely E-7 and E-12 directed towards a C-terminal epitope spanning amino acids 185–430, and H-6 specific for an epitope encompassing the amino acid sequence 251–283 internal to the FAMIN protein. In WB analyses of protein extracts from HeLa cells transfected with FAMIN or GFP-FAMIN expressing plasmids, all three antibodies gave rise to protein bands of the expected size (48 kDa for the wt and 75 kDa for the GFP-tagged; Fig 2A), while at the same time confirming the absence of endogenous protein product in this epithelial cell line. Best results were obtained with the anti-FAMIN E-12 antibody, which was therefore selected for the next series of experiments.

#### Characterization of FAMIN expression in THP-1 cells

**Fig 3:**
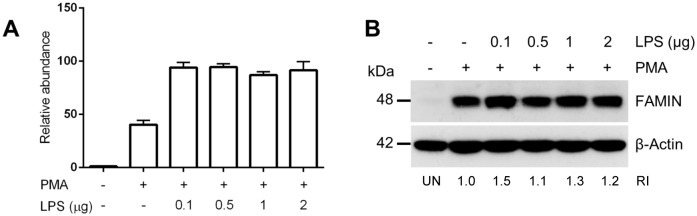
LPS stimulation of THP-1 cells results in FAMIN upregulation. (A) qRT-PCR analysis of *LACC1* mRNA expression in THP-1 cells stimulated with PMA and LPS. (B) WB analysis of FAMIN protein expression in THP-1 cells stimulated with PMA and LPS. Relative intensity (RI) from densitometric analysis is reported. Data are representative of three independent experiments. Abbreviation; UN: unstimulated control.

THP-1 cells can be differentiated into macrophages upon PMA treatment, hence we set out to test FAMIN protein expression under these conditions. As shown in Fig 3A and 3B, FAMIN expression was undetectable in untreated THP-1 cells, whereas PMA-induced differentiation resulted in strong upregulation of FAMIN. Having confirmed the pattern of protein expression in HeLa (negative) and THP-1 (positive) cell lines, we established an experimental model system where FAMIN expression could be abrogated via siRNA-mediated mRNA knockdown. For this purpose, we transfected THP-1 cells with a pool of 4 *LACC1*-directed siRNA for 48hrs (see methods), and used WB to analyze FAMIN expression, which was undetectable after siRNA-mediated knockdown (Fig 2B).

In order to verify the versatility of the experimental system, and the possibility to modulate FAMIN expression after THP-1 differentiation, we studied mRNA and protein expression under LPS stimulation at different concentrations. As shown in Fig 3A, *LACC1* mRNA expression was upregulated almost 50x upon PMA differentiation, compared to undifferentiated THP-1 cells, confirming previous protein expression data. LPS stimulation of differentiated THP-1 cells resulted in further increase of *LACC1* mRNA expression (Fig 3A), although this increase (2x compared to absence of LPS stimulation) was not amenable to detection in WB analysis (Fig 3B).

#### FAMIN expression in human white blood cells

**Fig 4:**
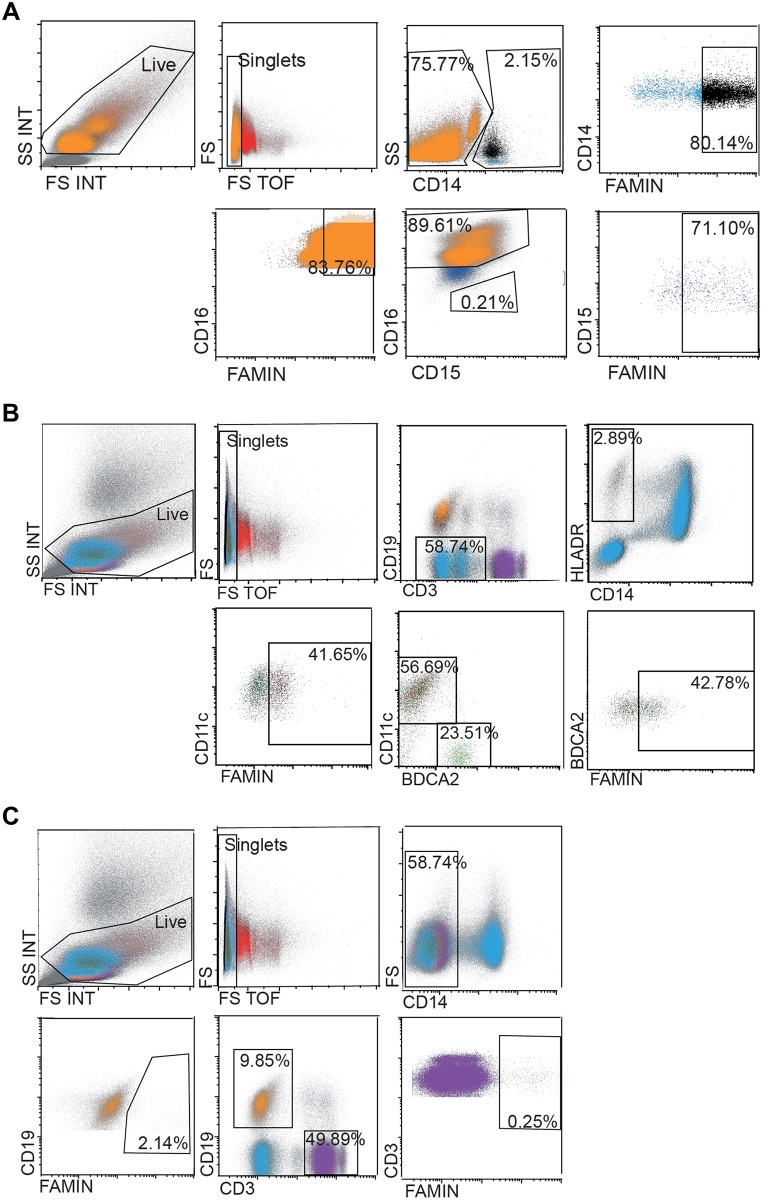
FACS analyses of FAMIN expression in PBMCs and granulocytes. PBMCs and granulocytes from human healthy donors were co-stained for different cell markers and FAMIN. Neutrophils, monocytes and DCs were shown to be FAMIN^+^. The events in the displayed graphs were first identified and gated by forward and side scatter parameters. (A) Gating strategy for FAMIN^+^ monocytes (CD14^+^, black) and CD14^-^ neutrophils (CD15^+^, purple and CD16^+^, orange). (B) Gating strategy for FAMIN^+^ myeloid DCs (CD11c^+^, brown) and plasmacytoid DCs (BDCA2^+^, green) was obtained from gating the CD3^-^CD19^-^ population followed by gating of CD14^-^HLADR^+^ cells. (C) Gating strategy for FAMIN^+^ T-cells (CD3^+^, purple) and B-cells (CD19^+^, orange). Numbers in the outlined areas indicate percent cells in each cell subtype. Data are representative of three independent experiments.

Next, we characterized the *LACC1* encoded protein FAMIN expression in primary cells, selecting human white blood cells (WBC) as the target population based on previous findings in macrophages and other immune cells. We first labeled the anti-FAMIN E-12 antibody with an Alexa Fluor^®^ 647 dye, in order to use it in FACS experiments. Cells obtained from buffy coats from healthy donors were co-stained with HLADR, CD14, CD11c, BCDA2, CD3 and CD19 antibodies to identify mononuclear (monocytes, dendritic cells, B and T cells, PBMCs) and CD14, CD3, CD19, CD15 and CD16 antibodies to identify polymorphonuclear cells (granulocytes) through FACS analysis. The results of this analysis are shown in Fig 4A–4C. We observed that 80% of the CD14^+^ monocytes, 71% of the CD15^+^ and 84% of the CD16^+^ neutrophils were positive for FAMIN. Furthermore, we also detected FAMIN expression in myeloid (CD11^+^, 42%) and plasmacytoid (BCDA2^+^, 43%) dendritic cells and virtually no expression in B-cells (CD19^+^, 2%) and T-cells (CD3^+^, 0.25%).

### FAMIN co-localizes with peroxisome markers

**Fig 5:**
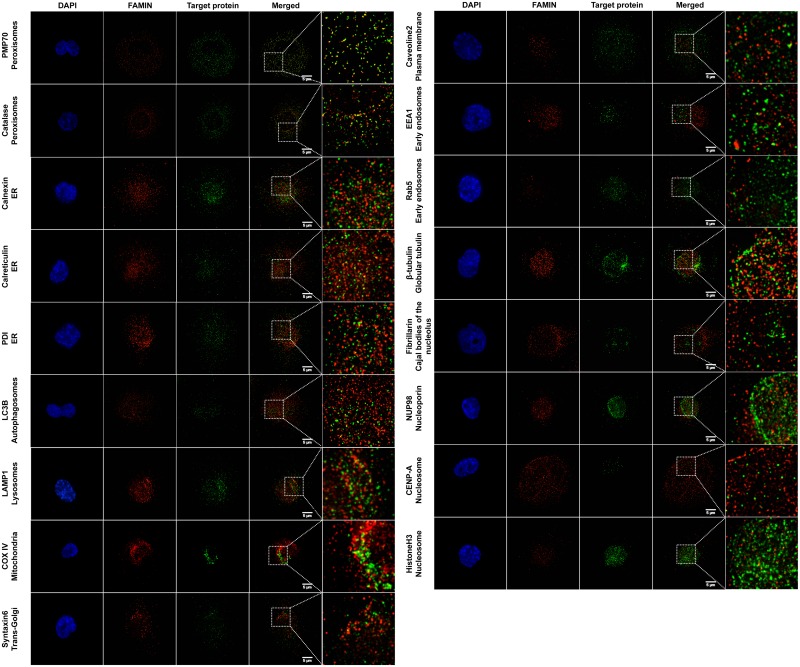
Characterization of FAMIN subcellular localization. PMA differentiated THP-1 cells were co-stained with a panel of antibodies directed towards different organelle markers and anti-FAMIN antibodies. Magnifications are shown for each staining on the right side of the pictures, together with indication of the target protein and corresponding compartment or cell organelle. Co-localization of FAMIN with PMP70 and catalase were previously shown and serve as positive control, Some additional co-localization could also be detected for endomembrane structure proteins detected at the level of endoplasmic reticulum (ER; calnexin, calreticulin and PDI), lysosomes (LAMP-1) and mitochondrion (COX-IV). Data are representative of six independent experiments.

We recently showed the subcellular localization of FAMIN at the level of peroxisomes (co-staining with the 70-kDa peroxisomal membrane protein (PMP70, also known as ATP-binding cassette sub-family D member 3; ABCD3) and catalase; CAT) and confirmed this co-localization through proximity ligation assay (PLA) [16]. Here we perform a thorough characterization of FAMIN expression in relation to several subcellular compartments and organelles, in order to verify the specificity of FAMIN localization to peroxisomes. For this experiment we used markers specific for different structural organelles such as fibrillarin for Cajal bodies of the nucleolus, β-tubulin for globular tubulin, NUP98 for nucleoporin, CENP-A and histone H3 for nucleosome and many sub-markers specific for endomembrane structures like LC3B for autophagosomes, EEA1 and Rab5 for early endosomes, calnexin, calreticulin and PDI for endoplasmic reticulum (ER), LAMP1 for lysosomes, COX-IV for mitochondrion, caveolin-2 for plasma membrane and syntaxin-6 for trans-Golgi. The results of these extensive immunofluorescence microscopy analyses in THP-1 cells are reported in Fig 5. FAMIN expression in THP-1 cells presented a relatively homogenous punctuate pattern and we confirmed the co-localization with the peroxisome markers PMP70 and catalase. In addition, some punctuate localization was also detected in correspondence of endomembrane structure proteins from the endoplasmic reticulum (calnexin, calreticuling and PDI), autophagosomes (LC3B), lysosomes (LAMP1), mitochondrion (COX-IV) and trans-Golgi (syntaxin-6).

### FAMIN does not exhibit laccase activity

**Fig 6:**
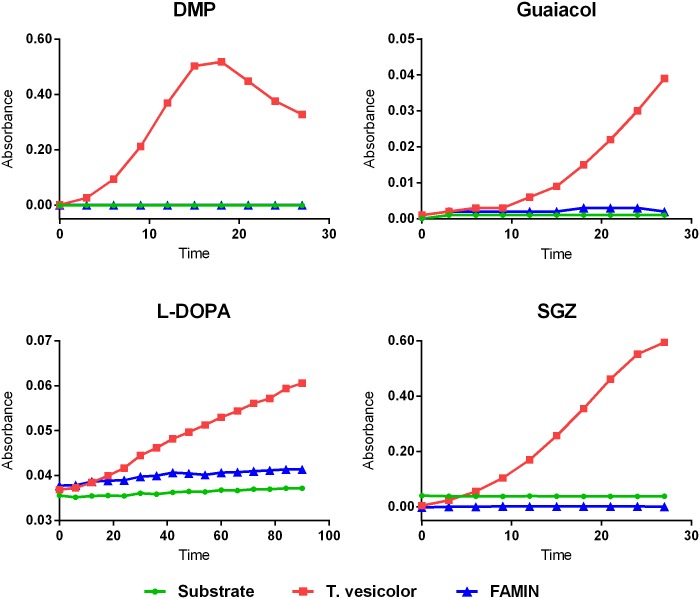
Analysis of human recombinant FAMIN laccase activity. Four phenolic substrates (DMP, Guaiacol, L-DOPA and SGZ) were tested in order to evaluate laccase activity of the C-terminal MYC/DDK-tagged recombinant FAMIN protein, as indicated. Data are representative of three independent experiments.

We have previously shown the lack of laccase activity for N- and C-terminal Strep-tagged FAMIN fusion constructs transfected, expressed, purified from HEK293T cells and tested on three substrates: a non-phenolic synthetic substrate (2,2-azinobis-(3-ethylbenzothiazoline-6-sulfonic acid), ABTS), a phenolic synthetic substrate (N,N-dimethyl-p-phenylenediamine, DMPPDA) and a naturally occurring laccase substrate (sinapic acid) [16]. Here we sought to further assess and confirm the absence of laccase properties of human FAMIN using a different FAMIN C-terminal MYC/DDK-tagged protein on four additional phenolic substrates; DMP, guaiacol, L-DOPA and SGZ (as positive control we used the laccase from *T*. *versicolor*). As shown in Fig 6, control oxidation of all four substrates could be detected, while the FAMIN recombinant protein failed to show similar laccase activity.

### PPAR ligands downregulate FAMIN expression

**Fig 7:**
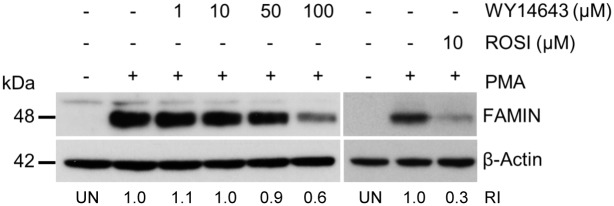
Effect of PPAR agonists FAMIN expression in THP-1 cells. WB analysis of FAMIN expression in THP-1 macrophages after 24hrs treatment with PPARα (WY14643) or PPARγ (rosiglitazone) as indicated. β-actin were used as a loading control. Relative intensity (RI) from densitometric analysis is reported. Data are representative of three independent experiments. Abbreviation; UN: unstimulated control.

In order to identify regulatory mechanisms controlling *LACC1* expression, we screened publicly available microarray data from 35 000 gene expression experiments using the search engine Genevestigator [18]. Looking across 2209 perturbations (stimulations) datasets, we obtained a list of 31 genes showing co-expression with *LACC1* in different conditions with correlation coefficient R ≥ 0.35 (Table 1). Gene-set enrichment analysis (GSEA) using this gene list and the Enrichr [19] online software was performed using the Kyoto Encyclopedia of Genes and Genomes (KEGG 2016) reference knowledgebase to infer biological pathway involvement. This resulted in the identification of PPAR signaling pathways, metabolic pathways, fatty acid biosynthesis, phagosomes, Jak-STAT signaling pathway and glycosaminoglycan biosynthesis as top enriched KEGG pathways (p-value ≤ 0.05) (Table 2). To experimentally corroborate this result, we tested the PPAR signaling pathway (top of the GSEA rank) for its potential to modulate FAMIN expression. PMA-differentiated THP-1 cells were treated with PPAR-α (WY14643) and PPAR-γ (rosiglitazone) ligands, and assessed on WB FAMIN protein expression in corresponding cell extracts. As shown in Fig 7, both ligands induced downregulation of FAMIN at 24hrs, and this appeared to be dose-dependent at least for WY14643, which was tested at different increasing concentrations.

**Table 1:** *LACC1* co-expressed genes.

Gene *	Ensembl alias	R correlation score
RIN2	Ras and Rab interactor 2	0.51
CTTNBP2NL	CTTNBP2 N-terminal like	0.45
STAM2	Signal transducing adaptor molecule 2	0.44
OSTM1	Osteopetrosis associated transmembrane protein 1	0.44
CLN5	Ceroid-lipofuscinosis, neuronal 5	0.44
IFNAR1	Interferon alpha and beta receptor subunit 1	0.44
SEC22B	SEC22 homolog B, vesicle trafficking protein	0.44
SGTB	Small glutamine rich tetratricopeptide repeat containing beta	0.44
STX12	Syntaxin 12	0.43
DSE	Dermatan sulfate epimerase	0.43
MYO5A	Myosin VA	0.42
AMPD3	Adenosine monophosphate deaminase 3	0.42
GPR34	G protein-coupled receptor 34	0.42
RAPH1	Ras association (RalGDS/AF-6) and pleckstrin homology domains 1	0.42
DOCK4	Dedicator of cytokinesis 4	0.41
RGL1	Ral guanine nucleotide dissociation stimulator like 1	0.41
TMOD3	Tropomodulin 3	0.41
230176-at	AFFY probeset 230176_at has probes which hit the genome in 11 locations	0.41
BLZF1	Basic leucine zipper nuclear factor 1	0.41
DCUN1D1	Defective in cullin neddylation 1 domain containing 1	0.40
SPRED1	Sprouty related EVH1 domain containing 1	0.40
HTATIP2	HIV-1 Tat interactive protein 2	0.40
240137-at	AFFY probeset 240137_at has probes which hit the genome in 11 locations	0.40
FCHO2	FCH domain only 2	0.40
ACSL6	Acyl-CoA synthetase long-chain family member 6	0.40
LACTB	Lactamase beta	0.40
GK	Glycerol kinase	0.36
SKIL	SKI-like proto-oncogene	0.36
KYNU	Kynureninase	0.35
GPR82	G protein-coupled receptor 82	0.35
PLA2G7	Phospholipase A2 group VII	0.35

* List of *LACC1* co-expressed genes (R ≥ 0.35) retrieved from Genevestigator.

**Table 2:** Pathways enriched with *LACC1* co-expressed genes.

KEGG 2016 gene-set enrichment analysis *	P-value
PPAR signaling pathway_Homo sapiens_hsa03320	0.0085
Metabolic pathways_Homo sapiens_hsa01100	0.0253
Fatty acid biosynthesis_Homo sapiens_hsa00061	0.0276
Phagosome_Homo sapiens_hsa04145	0.0374
Jak-STAT signaling pathway_Homo sapiens_hsa04630	0.0392
Glycosaminoglycan biosynthesis—chondroitin sulfate / dermatan sulfate_Homo sapiens_hsa00532	0.0411

* Pathways from the gene-set enrichment analysis on the list of *LACC1* co-expressed genes with a p-value ≤ 0.05.

**Fig 8:**
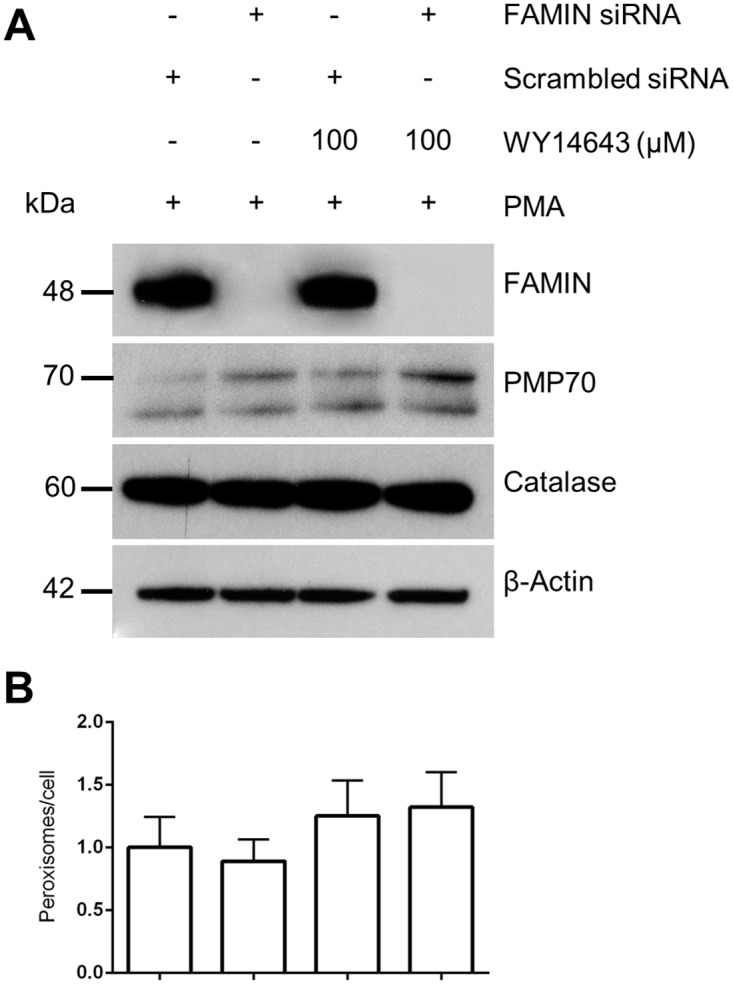
Effect of siRNA-mediated FAMIN knockdown on peroxisome number and peroxisome protein expression. (A) WB analysis of PMA-induced THP-1 macrophages show diminished FAMIN expression upon siRNA knockdown. Subsequent treatment with WY14643 does not affect peroxisome protein expression. (B) However, a possible trend towards increasing numbers of peroxisomes per cell in THP-1 macrophages could be observed after treatment with PPARα agonist WY14643, independent of FAMIN presence or absence. Data are representative of three independent experiments.

Finally, we asked whether ablation of FAMIN expression, by siRNA-mediated knockdown in THP-1 cells in the presence or absence of PPAR-α ligands (known to affect peroxisomes at least in murine model systems [21]), affects peroxisome number and peroxisome protein expression. As shown in Fig 8A and 8B, FAMIN knockdown did not affect peroxisome number, nor did it impact the expression level of peroxisome proteins PMP70 and catalase (lanes 1–2). Similarly, while WY14643 stimulation induced a slight increase in peroxisome number (Fig 8B), no differences were observed between control and siRNA-mediated FAMIN-depleted THP-1 cells (lanes 3–4).

## Discussion

Recently, we showed that the *LACC1* gene codes for a master regulator of macrophage immunometabolic function. Polymorphic variation in this gene strongly affects the risks of CD, UC, leprosy and JIA [4,6–13,16], hence efforts aimed at gaining increased understanding of its function and pathogenetic role are of relevance to immune-related diseases.

In agreement with previous evidence [16], we detect higher *LACC1* expression in spleen and lymph nodes, tissues where monocytes and macrophages are resident. We also show that FAMIN is expressed in human monocytes, myeloid and plasmacytoid dendritic cells and neutrophils, where its expression is detected at highest levels. Interestingly, this resembles the expression pattern of another gene, *protein tyrosine phosphatase non-receptor type 22* (*PTPN22*), which is also involved in the susceptibility to immune-mediated diseases, including JIA. PTPN22 removes phosphorylated tyrosine residues from target proteins, and tyrosine phosphorylation has been shown to be important in the regulation of neutrophil function [22]. This is highlighted by the observation of increased neutrophil effector functions, including ROS production, in human carriers of the rheumatoid arthritis (RA) risk allele R620W (rs2476601) [23]. Similarly, we have recently shown that macrophages and neutrophils from individuals carrying the hypomorphic *LACC1* variant Ile254Val (rs3764147) produce less ROS [16]. Interestingly, a SNP-SNP interaction for *PTPN22* (rs2476601) and *LACC1* (rs3764147) was shown to confer increased risk of UC in a study of Lithuanian and Latvian IBD patients [24], indicating that these two genes may act across overlapping pathogenetic mechanisms of disease predisposition, through neutrophils or macrophage-specific functions.

Ning et al. [25] recently performed an integrative approach to understand IBD risk effects due to CD genome-wide significant loci, and identified *LACC1* as one out of 213 prioritized genes among a total number of 1328 known CD-associated genes. In this study, the authors also explored differential gene expression in human blood-derived macrophage of the M1 (pro-inflammatory) and M2 (tissue repair) types and, consistent with our findings in THP-1 cells, FAMIN expression was shown to be modulated by LPS and IFNγ [25]. Although no data on human M1 macrophages were reported, we have showed higher levels of FAMIN in murine bone marrow-derived M1 compared to M0 and M2 macrophages in our previous study [16]. M1 macrophages have a pro-inflammatory phenotype, secreting cytokines that have been shown to be involved in the pathophysiology of IBD [26,27], and FAMIN-mediated triggering of the NLRP3 inflammasome does indeed induce secretion of these cytokines. Of note, absence of FAMIN has been associated with downregulation of the pro-inflammatory cytokines IL-1β, TNF and CXC1 in a knock-out mouse model [16].

In macrophages, FAMIN has been shown to control the bioenergetic capacity by promoting *de novo* lipogenesis (DNL) and FAO at peroxisomes [16], a cell site we also highlighted here through a thorough fluorescence microscopy analysis of co-localization experiments. In particular, FAMIN detection appears to be confined to peroxisomes and, to a lower extent, endomembrane structures like those found at the level of the endoplasmic reticulum. The latter localization may correspond to nascent FAMIN proteins detected before travel to peroxisomes, which would be consistent with the finding of unfolded sJIA and EOCD *LACC1* mutants (Cys284Arg) retained in the ER [16]. Peroxisomes require FAO to properly function, FAMIN-dependent FAO triggers inflammasome activation and ROS production [16], and some of the defense potential of macrophages and neutrophils is also exerted through the peroxisome content (antimicrobial peptides, peptidases and hydrolases) [28]. Maintaining peroxisome homeostasis in these phagocytic cells is therefore essential for the host defense, possibly reflected in the observed decrease of peroxisome numbers in intestinal epithelial cells tissues from CD patients, showing negative effects on FAO [29].

Our siRNA-mediated knockdown of FAMIN expression did not reveal any direct effect on peroxisome numbers. However, GSEA analysis of *LACC1* co-expressed genes highlighted the PPAR signaling pathway as the top enriched KEGG process. PPARs are transcription factors involved in the control of carbohydrate and lipid metabolism, but have also been shown to play important anti-inflammatory roles [21]. Activation of PPARs negatively regulates macrophage-induced inflammation by interfering with the NF-κB or AP1 signaling pathway [30]. Impaired expression of PPARγ has been detected in both UC and CD [31,32]. Hontecillas et al. [33] have shown in a mouse model of IBD that animals deficient in macrophage-specific PPARγ expression failed to recover after treatment with the PPARγ agonist pioglitazone. PPAR transcription factors are activated upon binding to their lipid-soluble natural and synthetic ligands [34]. In order to study the effect of PPAR activation on FAMIN expression we used the synthetic PPARα ligand WY14643 and the PPARγ ligand rosiglitazone. PPAR-ligand stimulation of THP-1 cells induced FAMIN downregulation (while still showing no difference between FAMIN^+^ and FAMIN^-^ PPAR-treated cells), suggesting the potential involvement of PPAR pathways in the modulation of *LACC1* expression. FAO is able to induce PPARs activation; hence one may speculate that the observed PPAR-driven downregulation of *LACC1* expression reflects a potential feedback-loop mechanism to control (FAMIN-dependent) FAO rate. At the same time, this may represent one genuine mechanism through which PPARs are able to modulate inflammation in macrophages or neutrophils, since downregulation of FAMIN may also result in reduced NLRP3 inflammasome activation and pro-inflammatory production of ROS. Of interest, it has been shown that M1 macrophages can be shifted to M2 phenotype by activation of the PPAR pathways [35]. Given that *LACC1* expression has been detected at higher levels at least in murine bone marrow-derived M1 compared to other macrophage subtypes [16], one may speculate that subtype switches may involve PPAR-driven changes in *LACC1* expression.

In summary, we report here confirmatory and novel results that contribute to further our understanding of *LACC1* function(s) and involvement in the pathogenesis of immune-mediated diseases. While the precise molecular mechanisms through which *LACC1* exerts its biological effects remains to be elucidated, these data expand the current knowledgebase by providing a resource of experimental conditions and investigative tools that may be exploited in future *LACC1* functional studies.
